# Expression of Tissue Remodeling- and Inflammation-Related Factors During the Wound-Healing Process in Humans

**DOI:** 10.3390/jpm15010014

**Published:** 2025-01-02

**Authors:** Dimitrios Vardakostas, Athanasios Moustogiannis, Zoe Garoufalia, Elli Karatza, Anastassios Philippou, Gregory Kouraklis, Michael Koutsilieris, Dimitrios Mantas

**Affiliations:** 1 Second Department of Propaedeutic Surgery, “Laiko” General Hospital of Athens “Laiko”, Medical School, National and Kapodistrian University of Athens, 11527 Athens, Greece; garoufz@ccf.org (Z.G.); ellikaratza@gmail.com (E.K.); gkouraklis@hotmail.com (G.K.); dvmantas@med.uoa.gr (D.M.); 2 Department of Physiology, Medical School, National and Kapodistrian University of Athens, 11527 Athens, Greece; amoustogiannis@nyc.gr (A.M.); tfilipou@med.uoa.gr (A.P.); mkoutsil@med.uoa.gr (M.K.)

**Keywords:** wound healing, TGF- β1, IL-6, TNF-α, uPA, uPA-r, MMP-2, MMP-9

## Abstract

**Background/Objectives**: There is a growing interest in the research of wound healing mechanisms worldwide. Particular attention has been paid to the expression of tissue remodeling- and inflammation-related factors. Herein, we investigate the expression patterns of TGF-β1, IL-6, TNF-a, uPA, uPA receptors, MMP-2, and MMP-9 through the four phases of the normal wound-healing process in humans. **Methods**: Twenty-two individuals presenting with a first episode of pilonidal sinus underwent surgical excision and the wound was left to heal by secondary intention. Sequential biopsies were collected on day 0 (operation), day 2 (inflammatory phase), day 9 (proliferative phase), and day 14 (tissue remodeling phase). The expression levels of the proteins were evaluated using reverse transcription–quantitative PCR. Statistical analyses were performed using GraphPad Prism software. One-way analysis of variance (ANOVA) with Dunn’s Multiple Comparison post hoc test was used. **Results**: The results showed statistically significant differences in the expressions of the factors during wound healing (*p* < 0.05). TGF-b1 increased on days 2 and 9. TNF-a increased on day 2 and then decreased on day 9. Il-6 was increased on day 2 and decreased on days 9 and 14. uPa mRNA increased up to day 9 but its receptor exhibited high expression throughout the observation time. Finally, MMP-2 mRNA expression increased on day 2 and declined on days 9 and 14, while MMP-9 was highly expressed until the 14th postoperative day. **Conclusions**: Each factor investigated in this study has an important and distinct role in the normal wound repair procedure. Further investigation is required to evaluate the tissue-specific regulation of these factors and their potential use as therapeutic targets or prognostic biomarkers in wound healing.

## 1. Introduction

Injuries are one of the most vital medical conditions that humans have tried to face, and many remedies, accompanied by their related theories, have been developed. Nowadays, in the era of modern medical research, although a tremendous number of scientific papers have been published, there is still a growing interest in wound healing among a wide range of researchers and health professionals.

Based on the established knowledge of wound-healing physiology, the wound-healing process is characterized by four consecutive, although overlapping, stages: (1) hemostasis, (2) inflammation, (3) proliferation, and (4) remodeling [1,2]. The hemostatic process commences as soon as blood vessels are ruptured and lasts from minutes to hours. At this point, while platelets are aggregated in order to control bleeding, biologically active molecules and growth factors, such as insulin-like growth factor I (IGF-I), transforming growth factor-β (TGF-β), Epidermal Growth Factor (EGF), Fibroblast Growth Factor (FGF), Vascular Endothelial Growth Factor (VEGF), and Platelet-derived Growth Factor (PDGF) are produced, influencing the forthcoming inflammatory response [3,4,5]. Acute inflammation is considered to last approximately three days. Specifically, the inflammatory response to wounding depends on a balance between inflammatory (Interleukin-1, Interleukin-6, Tumor Necrosis Factor-a) and anti-inflammatory (Interleukin-4, Interleukin-10) cytokines, leading, most of the time, to successful healing without excessive responses [6].

Immune cells, fibroblasts, and keratinocytes are attracted chemotactically to the wound site, while macrophage-derived cytokines activate and attract fibroblasts to the wound site as well [7,8]. Neutrophils are the first responders during the inflammatory phase, clearing pathogens and debris [9]. Macrophages play a pivotal role in both the inflammatory and resolution phases, thanks to their ability to polarize into two distinct phenotypes: M1 (pro-inflammatory) and M2 (anti-inflammatory and tissue repair) [10].

After 48–72 h of tissue damage, the proliferation phase begins. Fibroblasts create a temporary scaffold with procollagen, hyaluronic acid, elastin, and proteoglycans, permitting neovascularization [11]. Endothelial cells also play a vital role in restoring blood supply and the keratinocytes migrate, proliferate, and differentiate to cover the wound, re-establishing the skin barrier.

When the wound defect is closed, remodeling can last for several months until the overall cell population declines, the inflammatory infiltrate resolves, the new vascular network is reorganized, and collagen type III is replaced by collagen type I [12,13,14]. Apart from the cell–cell and cell–extracellular matrix interactions, all wound-healing phases are regulated by many growth factors, cytokines, and proteinases [15]. Particular attention has been paid to the components of the bioregulatory system of TGF-β1, uPA, and MMPs and their role not only in the normal wound-healing process, but also in abnormal scars; nevertheless, their role is not yet fully elucidated. In this study, we investigated the expression patterns of TGF-β1, IL-6, TNF-a, uPA, uPA-r, MMP-2, and MMP-9 through the four phases of normal wound healing. The characterization of the expression profile of these factors throughout the wound-healing process could contribute to a better understanding of wound-healing physiology, thus helping to improve wound care and prevent inefficient healing, hypertrophic scars, and keloids.

## 2. Materials and Methods

Ethical approval. Written informed consent was obtained from all volunteers participating in this study, which was approved by the Local Ethics Committee (“Laiko” General Hospital of Athens, protocol code 263, and date of approval 03/13/2017). All experimental procedures adhered to the principles outlined in the Declaration of Helsinki.

Subjects and sample collection. Thirty healthy individuals suffering from pilonidal sinus underwent surgical excision under local anesthetic and the wound was left to heal by secondary intention. At the beginning of the operation, local anesthesia was achieved by injecting lidocaine 2%. A sharp scalpel was used for the excision of the affected area and a diathermy for hemostasis. Macroscopically, the incisional margins were free of inflammation. In the following days, the wounds were treated daily using only normal saline, sterile gauze, and common self-adhesive patches. Sequential biopsies were obtained from the open surgical wounds at the time point of excision (day 0), at the phase of inflammation (day 2), at the phase of proliferation (day 9), and finally at the phase of remodeling (day 14). It was decided not to collect samples from intact skin to prevent any harm to the subjects. The samples were collected from the wound sores, measured 0.5 cm in depth, and consisted of full-thickness biopsies of the wound, including both skin and subcutaneous tissue. Tissue samples were immediately immersed in RNAlater stabilization solution (Ambion RNAlater, Thermo Fisher Scientific Inc., Waltham, MA, USA) and stored frozen at −80 °C. In 22 cases, all four sequential tissue samples were collected, whereas in 8 cases, at least one sample was not obtained due to defective follow-up or wound contamination.

### 2.1. RNA Extraction

RNA was extracted from all tissue samples through cell lysis using NucleoZOL (Mecherey-Nagel, Düren, Germany). Total RNA was isolated from the lysates following the manufacturer’s protocol. The extracted RNA was dissolved in RNase-free water (Invitrogen, Carlsbad, CA, USA), and its concentration and purity were assessed spectrophotometrically using a Thermo Nanodrop 2000 (Thermo Scientific™, Waltham, MA, USA) by measuring absorbance at 260 and 280 nm. RNA integrity was verified by inspecting the electrophoretic patterns of 18S and 28S ribosomal RNA on ethidium bromide-stained 1% agarose gels under UV light. The total RNA extracts were stored at –80 °C until further analysis, which involved determining the mRNA levels of target genes using reverse transcription and semi-quantitative real-time PCR.

### 2.2. Reverse Transcription and Real-Time Polymerase Chain Reaction (PCR)

Total RNA from each tissue sample was used to synthesize single-stranded cDNA through reverse transcription using ProtoScript II reverse transcriptase (NEB, Ipswich, MA, USA). For reverse transcription, 1 μg of total RNA was mixed with 300 ng of random primers, 300 ng of oligo(dT)23VN primers, and nuclease-free water in a final volume of 8 μL. The mixture was heated at 65 °C for 5 min and immediately placed on ice. Subsequently, 10 μL of ProtoScript II Reaction Mix and 2 μL of ProtoScript II Enzyme Mix were added, and the samples were incubated at 25 °C for 5 min, followed by 45 °C for 1 h, as per the manufacturer’s instructions. The reaction was terminated by heating to 80 °C for 5 min, and the cDNA was stored at –20 °C.

Real-time PCR was conducted using a Bio-Rad iCycler thermal cycler (iQ5 Real-Time PCR Detection System, Hercules, CA, USA) with iQ™ SYBR Green Supermix reagents (Bio-Rad). Specific primer sequences for detecting IL-6, TNF-α, TGF-β1, uPA, uPA r, MMP-2, and MMP-9 are listed in Table 1. To avoid genomic DNA contamination, primers were designed across exon boundaries. Each 20 μL PCR mixture included 50 ng of cDNA, 12.5 μL SYBR Green Master Mix, 0.4 μM of each primer, and nuclease-free water. The thermal cycling parameters were as follows: an initial denaturation step at 95 °C for 4 min, followed by 45 cycles of 95 °C for 12 s, 61 °C for 30 s (annealing), and 72 °C for 30 s (extension). A final extension was performed at 72 °C for 5 min.

Gene expression levels were quantified by calculating the threshold cycle (Ct), defined as the number of cycles required for fluorescence to exceed the detection threshold. GAPDH served as a housekeeping gene for normalization (relative quantification, ΔCt). Each sample was analyzed in duplicate, and the data were averaged.

Melting curve analysis was performed after the final cycle, with fluorescence monitored from 70 °C to 95 °C, to confirm the specificity of amplification. Each gene-specific primer yielded a single melting peak, indicating specificity, which was further verified by electrophoretic analysis of the PCR products. Negative controls included reactions without cDNA and without template.

### 2.3. Statistical Analysis

Statistical analysis was performed using one-way analysis of variance (ANOVA) followed by Dunn’s Multiple Comparison post hoc test, with GraphPad Prism 5 software. All experiments were conducted in duplicate, and the results are expressed as mean ± standard error of the mean (SEM). A *p*-value of <0.05 was considered statistically significant.

## 3. Results

### 3.1. Transforming Growth Factor-β1

**Figure 1:**
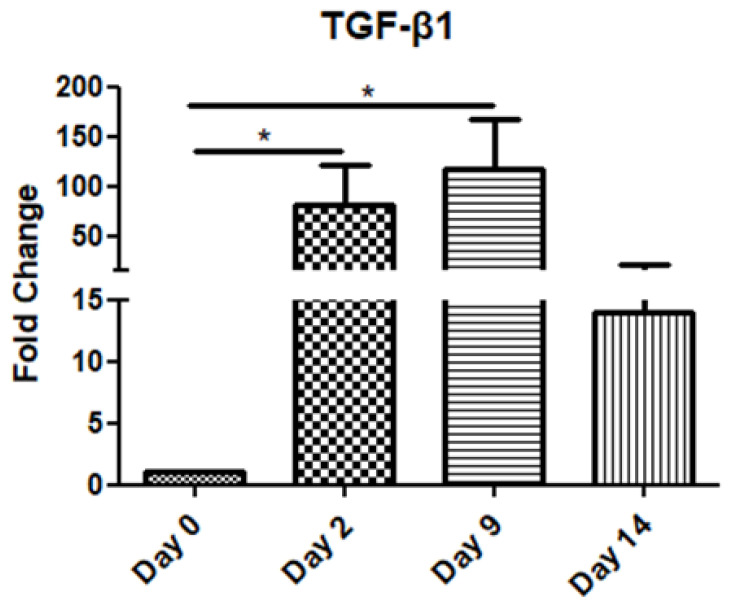
Expression pattern of transforming growth factor-β1 (TGF-β1) at four time points of the wound-healing process. Quantitative analysis of TGF-β1 mRNA expression in tissue samples from wound sores of pilonidal excisions compared to control (day 0 of the operation). The mRNA values were normalized to the corresponding GAPDH mRNA expression and are expressed as fold changes compared to day 0 (day of operation). *: Significantly different, *p* < 0.05.

The TGF-β1 mRNA levels increased gradually on day 2 and day 9 compared to the day of operation (day 0), before decreasing on day 14 (Figure 1).

### 3.2. Inflammatory Cytokines

**Figure 2:**
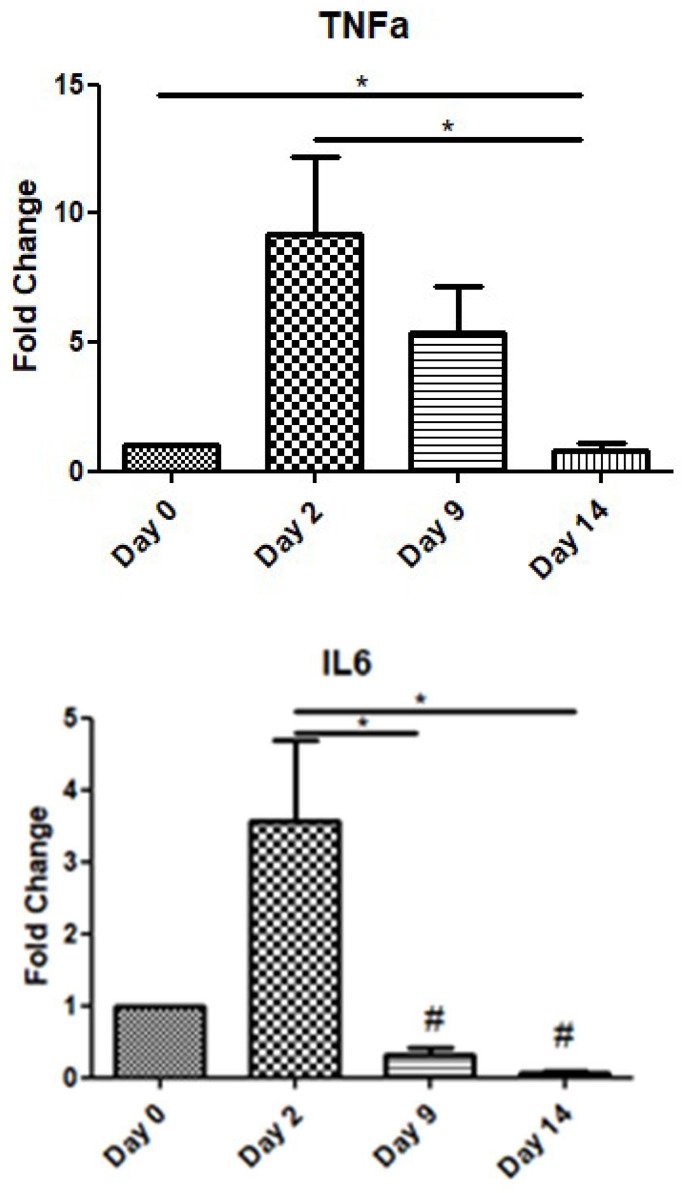
Expression patterns of inflammatory cytokines at four time points of the wound-healing process. Quantitative analysis of TNF-a and IL-6 mRNA expression in tissue samples from wound sores of pilonidal excisions compared to day 0 (day of the operation). The mRNA levels were normalized to the corresponding GAPDH mRNA and are expressed as fold changes compared to day 0. *: Significantly different, *p* < 0.05; #: significantly different compared to day 0; *p* < 0.05.

In order to assess the role of inflammatory cytokines during the wound-healing process, we examined the expression levels of TNF-a and IL-6 over time. TNF-a showed a peak increase on day 2, before gradually decreasing on day 9, while on day 14, its expression level was below that at day 0. Similarly, IL-6 also exhibited an increase on day 2, before decreasing on days 9 and 14 below the expression levels of day 0 (day of operation) (Figure 2).

### 3.3. Urokinase-Type Plasminogen Activator (uPA)/uPA Receptor(uPAR)

**Figure 3:**
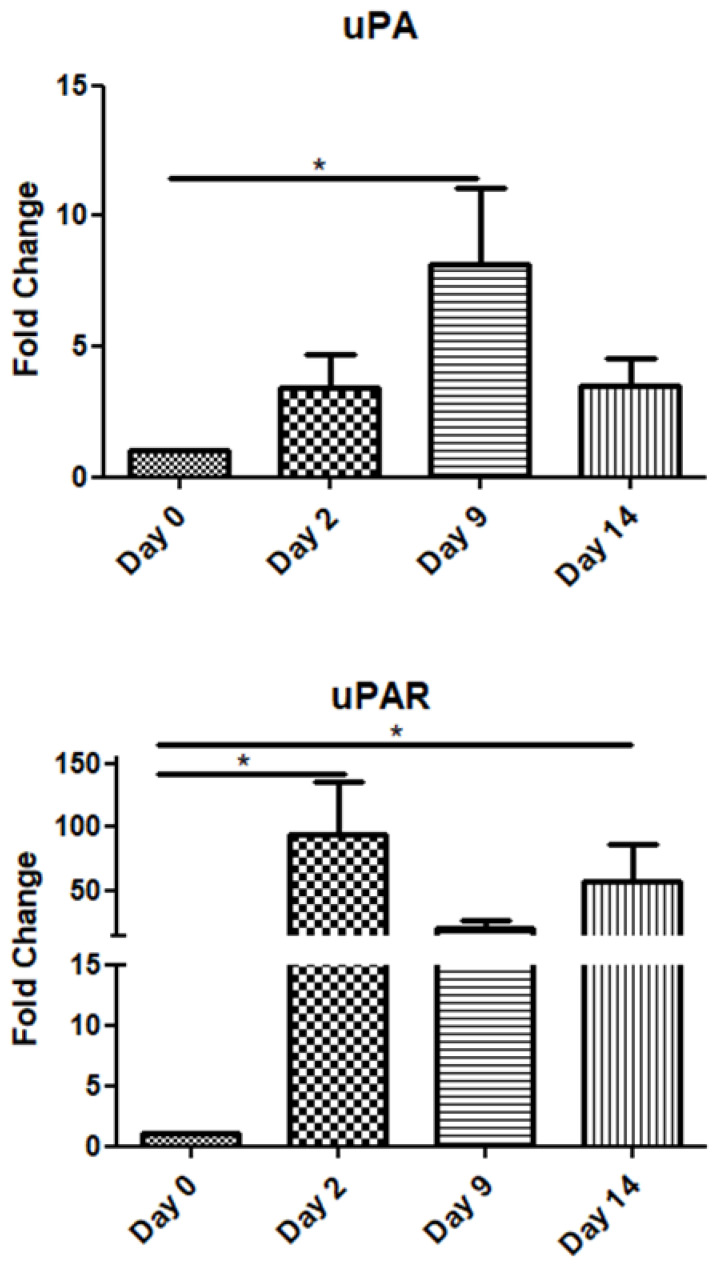
Expression patterns of uPA/uPAR at four time points of the wound-healing process. Quantitative analysis of uPA and uPAR mRNA expression in tissue samples from wound sores of pilonidal excisions compared to day 0 (day of the operation). The mRNA values were normalized to the corresponding GAPDH mRNA expression and are expressed as fold changes compared to day 0. *: Significantly different, *p* < 0.05.

The uPA mRNA expression gradually increased up to day 9 and declined on day 14. Interestingly, its receptor (uPAR) exhibited high expression throughout the observation period, i.e., up to day 14 post-operation (Figure 3).

### 3.4. Matrix Metalloproteinases

**Figure 4:**
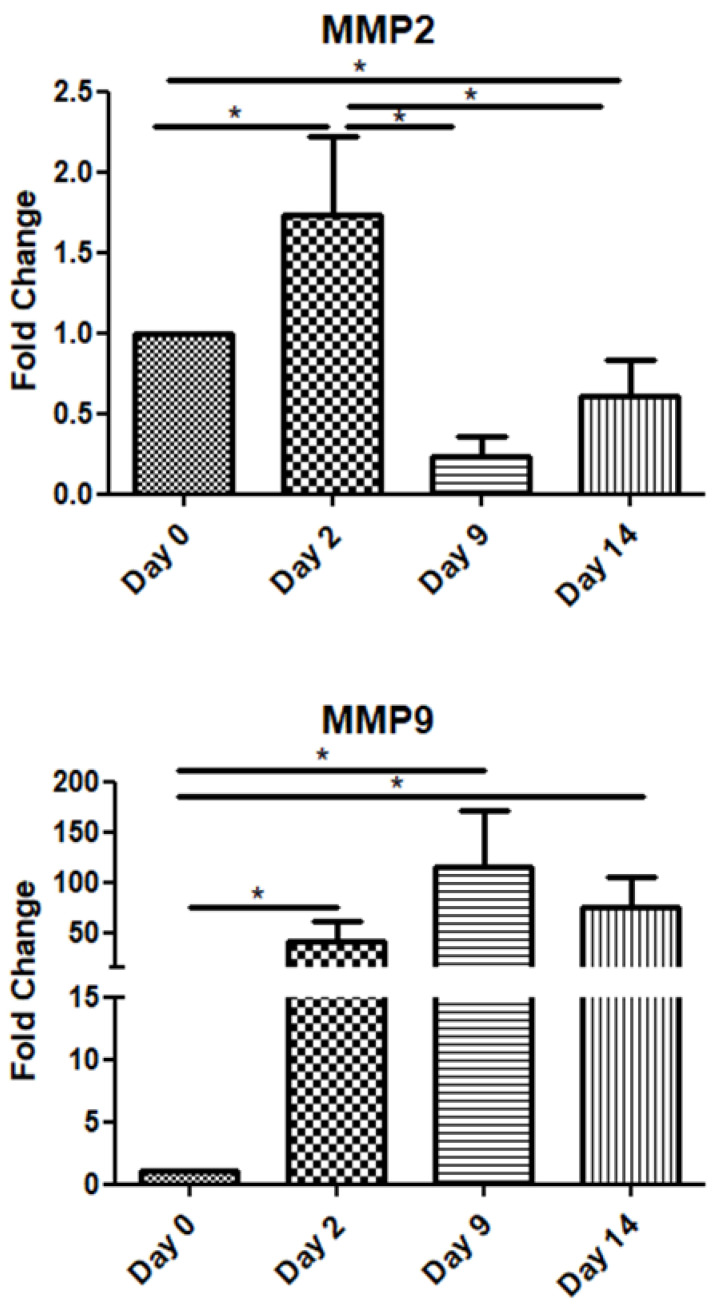
Expression patterns of matrix metalloproteinases at four time points of the wound-healing process. Quantitative analysis of MMP-2 and MMP-9 mRNA expression in tissue samples from wound sores of pilonidal excisions compared to day 0 (day of the operation). The mRNA values were normalized to the corresponding GAPDH mRNA levels and are expressed as fold changes compared to day 0. *: Significantly different, *p* < 0.05.

Among the metalloproteinase family members, in this study, we examined two of the most representative ones, namely MMP-2 and MMP-9. The MMP2 mRNA expression increased on day 2 and declined on days 9 and 14. Interestingly, MMP-9 exhibited high expression throughout the observation period, i.e., up to day 14 post-operation (Figure 4).

## 4. Discussion

Although many studies have been conducted aiming to reveal the regulatory factors and pathways of wound healing, most of them use animal models or in vitro techniques [16,17]. Thus, due to the lack of related research in humans [18], the present study was designed to determine the expression patterns of some of the most pivotal key regulators of healing, namely TGF-β1, TNF-a, IL-6, uPA, uPAR, MMP-2, and MMP-9, during the normal three-stage tissue repair of open sacrococcygeal wounds in healthy individuals. The expression patterns of key mediators during wound healing reveal distinct temporal dynamics that align with the different phases of the process, potentially reflecting their biological roles in the healing process. These factors could serve as biomarkers for assessing wound healing and predicting its progression [19,20,21], while also presenting potential targets for therapeutic intervention [22,23].

TGF-β1 mRNA expression demonstrated a significant increase during the inflammatory stage (day 2), continuing to rise throughout the proliferation phase (up to day 9), before decreasing in the remodeling stage (day 14). This trend underscores TGF-β1′s critical role in orchestrating inflammation, promoting tissue repair, and later supporting ECM remodeling.

TGF-β1 is considered to have a very broad spectrum of actions and affects all cell types involved in the process of wound healing [24,25]. It belongs to the TGF-β family (TGF-β1, 2, and 3) and it is initially secreted as a latent form. When activated, its function is exerted via type I or II transmembrane serine/threonine kinase receptors and its intracellular signal is mainly mediated by Smad proteins [15,26]. As a result, extracellular matrix molecule and metalloproteinase production, cell migration, and wound contraction are stimulated by TGF-β1 [27], serving both as a pro-inflammatory and fibrotic factor. More specifically, immediately after tissue injury, TGF-β1 is upregulated and produced by platelets, macrophages, and keratinocytes in order to initiate inflammation and granulation [28,29]. This combination of different cellular sources and temporary storage ensures a continuous supply of TGF-β1 throughout the repair process. In our study, as in others [24,30,31], a rapid induction of TGF-β1 was observed. Later, at the proliferation stage, its expression increased and, although more mildly, was sustained during the remodeling stage. In another experimental study on cutaneous healing, it was observed that endogenous TGF-β1 increased rapidly after the full-thickness injury and reached a peak level on the third day, that is, at the peak of the inflammatory phase [32].

TNF-α expression peaked on day 2, reflecting its involvement in the early inflammatory response, but it declined by half on day 9 and returned to preoperative levels by day 14. Similarly, IL-6 showed a notable increase only during the early inflammatory phase (day 2), with subsequent levels on days 9 and 14 falling below the baseline observed on day 0. These trends highlight the transient nature of these cytokines during the resolution of inflammation. The important role of pro-inflammatory cytokines IL-6 and TNF-a in wound repair has been validated by several experimental studies [33,34,35,36]. The various effects of these cytokines on the healing process include chemotaxis and the proliferation of keratinocytes and fibroblasts, the synthesis and degradation of ECM proteins, and the regulation of the local immune response [37]. Neutrophils and macrophages are the main source of these cytokines, but other resident cell types are also implicated in their secretion [38]. The pro-inflammatory role of these cytokines is strongly supported by the intense upregulation of their expression during the inflammatory phase [39,40].

uPA mRNA expression showed a significant increase on day 9, correlating with its role in ECM degradation and cell migration during the proliferation phase. Its receptor (uPAR), however, remained consistently expressed throughout the observation period, suggesting its ongoing importance in wound-healing processes. Urokinase-type plasminogen activator (uPA) is an extracellular serine protease that, when bound to its receptor (uPAR), converts plasminogen into plasmin. uPA, matrix metalloproteinases (MMPs), and plasmin, the primary fibrinolytic enzyme, are involved in various non-fibrinolytic processes, including extracellular matrix degradation, remodeling, and cellular rearrangement. Plasminogen activators and MMPs work synergistically to regulate growth factor activities, such as hydrolyzing insulin-like growth factor-binding proteins (IGFBPs) and activating latent transforming growth factor-beta (TGF-βs) [41,42,43]. Tissue hypoxia, a common feature within wounds, stimulates uPA production. Stavropoulou et al. investigated the expression of uPA, uPAR, and TGF-β1 during the early and late phases following myocardial infarction (MI) in rats [44]. Their findings revealed a significant upregulation of uPA and uPAR at both transcriptional and protein levels during the early post-MI phase, with uPA expression peaking at 1 h and uPAR at 24 h post-infarction.

Finally, the expression patterns of matrix metalloproteinases (MMPs) also varied over time. MMP-2 mRNA levels peaked on day 2 before declining, while MMP-9 maintained high expression throughout the 14-day postoperative period, indicating its extended role in matrix remodeling and tissue repair. MMPs are a structurally related family of enzymes that have the ability to degrade nearly all extracellular matrix components and consequently determine the composition and structure of extracellular matrix [45]. Soo et al. tried to characterize the temporal changes in the mRNA expression profiles of MMPs during the phases of excisional skin repair in rats [46]. MMP-2 mRNA was moderately expressed in nonwounded specimens and did not increase significantly until 3 days after wounding. Peak expression was attained on day 5, declined slightly from days 5 to 7, and remained significantly higher than baseline until day 14. On the other hand, MMP-9 had minimally detectable levels at the beginning but within 12 h after wounding exhibited significantly large increases in mRNA levels. MMP-9 showed peak levels between 24 h and 3 days and subsequently declined to near baseline by day 14.

In the context of investigating the role of poly ADP-ribosylation (PARylation) in a mouse model of excision wounding, El-Hamoly et al. also determined the mRNA expression of IL-6, TNF-a, and MMP-9 in homogenates of wounds from wild-type mice [47]. Expression of MMP-9 increased until day 4 but declined by day 8. Levels of IL-6 and TNF-a mRNA increased on day 2 after wounding and declined by day 8 [47]. Mirza and Koh isolated cells directly from mouse wounds and measured the cytokine release from these cells ex vivo on the 5th and 10th days of the experimental period. It turned out that the total release of TGF-β1 from all the cell subsets was large enough at the inflammatory phase and it doubled at the proliferative phase. On the other hand, TNF-a and IL-6 were found to be released in a great amount on the 5th day but by the end of the 10th day, their quantity had diminished [48].

Putting all these findings together, it becomes clear that each factor examined in this study has an important and distinct role in the fine-tuned machine of normal wound repair. Directly after the damaging tissue insult, all the involved cells in the vicinity promptly deliver a great amount of TGF-β1 to intensify the inflammation, recruit other cells, and stimulate extracellular matrix reconstruction and fibrosis. The presence of TGF-β1 is required throughout the procedure, although at later stages, its quantity is decreased. As expected, the inflammatory phase is characterized by the upregulation of the pro-inflammatory cytokines IL-6 and TNF-a. Inflammation is very important both for the sterilization of trauma and the promotion of healing. However, its influence must be restricted at the stages of proliferation and remodeling, avoiding perpetuated inflammatory processes, impaired healing, and scarring. Urokinase-type plasminogen activator is well known for its function in clot lysis. The findings of the present study suggest that uPA, along with its receptor, also intertwines with other factors, such as TGF-β1 and MMPs, and their elevated expression is beneficial during healing. We believe that the gradual increase in uPA mRNA expression observed up to day 9 suggests that its production is initiated after clot formation and the inflammatory phase. Its peak activity appears to be necessary just before the onset of the remodeling phase, having already triggered a cascade of downstream processes. In normal conditions, uPA receptor expression remains dormant in healthy tissues but becomes highly upregulated during cell migration and inflammation [49]. Interestingly, its protease ligand or proteolytic function is not always required [50]. For instance, the binding of the inactive proenzyme form (pro-uPA) to uPAR can also initiate plasmin activation [51]. Additionally, intracellular signaling can be mediated through other receptors such as vitronectin and integrins [52]. This indicates that uPAR’s role extends beyond uPA binding, emphasizing its continuous availability during the wound-healing process. Lastly, MMPs must be expressed at an optimum time and level to permit an uneventful restoration of the sore [53].

Surprisingly, some studies have shown partly controversial data concerning the effects of some factors, e.g., TGF-β1 [15,27]. However, such confusing results reflect the complex nature of the biological interplay between these functions, which may be specific to cell types and conditions [54].

The absence of a control group in our study is a substantial limitation. Moreover, the relatively small number of participants is also another limitation of the study, along with the fact that no standardized method for the estimation of the healing progress of the wound was applied. In addition, future similar studies should be enriched with histological assays and immunoblotting analyses.

## 5. Conclusions

In conclusion, the present study characterized the expression patterns of various tissue remodeling- and inflammation-associated factors throughout the wound-healing process. These observations collectively emphasize the distinct temporal roles of these mediators in coordinating the progression of wound healing from inflammation to proliferation and, finally, to remodeling. Thus, this study can be a resource to be used as a database providing evidence derived from human tissues regarding the implication of the examined factors in wound healing. The possible tissue-specific regulation of these factors in the pathophysiology of wound healing requires further investigation, to confirm whether they could be used as potential therapeutic targets and/or prognostic biomarkers in wound healing and/or wound age.

## Figures and Tables

**Table 1:** Sequences of the primers used for RT-qPCR.

Target Gene	5′-3′ Forward Primer Sequence	5′-3′ Reverse Primer Sequence
GAPDH	*TCA AGA ACG AAA GGA GG*	*GGA CAT CTA AGG GCA TCA CA*
UPA	*GTC TAC CTG GGT CCC TCA*	*CAC AGC ATT TTG GTG GTG AC*
TGF-β1	*ACC TCG GCT GGA ACT GGA TC*	*GAT CAT GTT GGA CAG CTG CTC C*
UPAR	*AGG GCC TGC GGT GCA TA*	*ACA GGC CTC TGG TCA CCA CCT*
IL-6	*ATG AAC TGG TTC TCC ACA AGC GC*	*GAA GAG CCC TCA GGC TGG ACTG*
TNF-a	*TCC TTC AGA CAC CCT CAA CC*	*AGG CCC CAG TTT GAA TTC TT*
MMPs-2	*CTT CCT AGG CTG GTC CTT ACT GA*	*CTG AGA CCT GAA GAG CTA AAG AGCT*
MMPs-9	*ATC CAG TTT GGT GTC GCG GAGC*	*GAA GGG GAA GAC GCA CAG CT*

## Data Availability

Dataset available on request from the authors.
